# Klotho Regulates Club Cell Senescence and Differentiation in Chronic Obstructive Pulmonary Disease

**DOI:** 10.1111/cpr.70000

**Published:** 2025-02-10

**Authors:** Min Li, Bo Chen, Sibo Sun, Kai Wang, Yu Wang, Jianqing Wu

**Affiliations:** ^1^ Department of Geriatrics, Jiangsu Province Hospital The First Affiliated Hospital of Nanjing Medical University Nanjing China; ^2^ Jiangxi Provincial Key Laboratory of Respiratory Diseases, Jiangxi Institute of Respiratory Diseases, The Department of Respiratory and Critical Care Medicine, The First Affiliated Hospital Jiangxi Medical College, Nanchang University Nanchang China; ^3^ China‐Japan Friendship Jiangxi Hospital National Regional Center for Respiratory Medicine Nanchang China; ^4^ Jiangsu Provincial Key Laboratory of Gerontology & Geriatrics Nanjing China; ^5^ Jiangsu Provincial Innovation Center of Gerontology & Geriatrics Nanjing China; ^6^ Department of Respiratory, SuQian Branch of Jiangsu Province Hospital The First Affiliated Hospital of Nanjing Medical University Suqian China; ^7^ Department of Geriatrics The Fourth Affiliated Hospital of Nanjing Medical University Nanjing China

**Keywords:** chronic obstructive pulmonary disease, club cell, klotho, scRNA‐seq, senescence

## Abstract

Chronic obstructive pulmonary disease (COPD) is characterised by chronic inflammation and senescence. Previous studies showed that club cells and club cell secretory proteins (CCSP) have anti‐inflammatory roles, which reduced in COPD. Klotho (KL) decreased in human COPD lung tissue. KL‐deficient mice showed aging phenotypes, such as obvious emphysema and premature senility at the early stage, which are characteristics of COPD. However, little is known about the relationship between KL, club cells, and COPD. We speculated lack of KL would aggravate club cell senescence, which contributes to COPD inflammation. We collected COPD lung tissue using single‐cell RNA sequencing (scRNA‐seq), revealing club cells heterogeneity and cellular senescence in COPD. In addition, KL and CCSP expressions were downregulated in cigarette smoke (CS)‐induced COPD mice, associated with increasing age‐related markers. After KL knockout, more ciliated cells appeared where club cells disappeared. Furthermore, KL deficiency aggravated club cell senescence and CSE‐induced pulmonary inflammation. To investigate the specific regulation mechanism, hnRNPA2/B1 was recognised and identified it was the key molecule in KL‐regulated club cell senescence, and neddylation of club cell was a crucial factor contributing to hnRNPA2/B1 downregulation. In vitro, SA‐β‐gal staining suggested the aging phenotype was aggravated in hnRNPA2/B1‐silenced groups, and hnRNPA2/B1 over‐expressed achieved a rescue result. Thus, KL could regulate club cell senescence and differentiation. When CS stimulates the small airway epithelium, KL deficiency aggravates lung inflammation, club cell senescence and dysfunctional of ciliated cell. Targeting neddylation might be a promising strategy to reverse lung aging and club cell senescence. These results provide a mechanism about COPD‐linked lung inflammation.

## Graphical abstract

We speculated lack of Klotho (KL) would aggravate club cell senescence, which contributes to COPD inflammation. In this study, we collected chronic obstructive pulmonary disease (COPD) lung tissue using single‐cell RNA sequencing (scRNA‐seq), revealing club cell heterogeneity and cellular senescence in COPD. Club cells were divided into four subpopulations, in addition, KL and club cell secretory proteins (CCSP) were downregulated in cigarette smoke (CS)‐induced COPD mice, associated with increasing age‐related markers. After KL knockout, more ciliated cells appeared where club cells disappeared, and resulting CCSP downregulation. Furthermore, KL deficiency aggravated club cell senescence and CSE‐induced pulmonary inflammation. To investigate the specific regulation mechanism, hnRNPA2/B1 was recognised and identified it was the key molecule in KL‐regulated club cell senescence, and neddylation of club cell was a crucial factor contributing to hnRNPA2/B1 downregulation. Thus, we concluded that KL could regulate club cell senescence and differentiation. When CS stimulates the small airway epithelium, KL deficiency aggravates lung inflammation, club cell senescence and dysfunctional of ciliated cell. Targeting neddylation might be a promising strategy to reverse lung aging and club cell senescence. This study describes a novel mechanism about COPD‐linked lung inflammation.

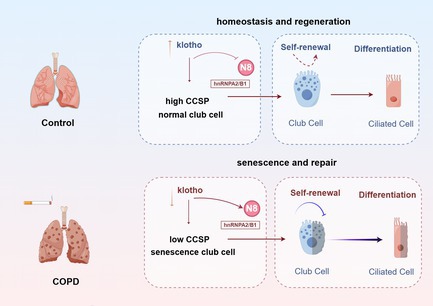

## 1 Introduction

Chronic obstructive pulmonary disease (COPD) is characterised by high morbidity, mortality, and disabilities [1]. In 2022, the Lancet divided COPD into five subtypes based on various trigger elements and recognised COPD as a heterogeneous disease [2]. Convincing evidence confirmed airway epithelium senescence as a pathogenic driver in COPD development, however, the specific mechanism underlying their association remains unknown.

The hallmarks of aging lung are reduced in lung function, airway mucociliary clearance, ciliary beat and enlarged alveoli [3, 4]. Ilias et al. [5] identified ciliated cells related genes were upregulated in older mouse lung tissue. Additionally, Elina et al. [6, 7] discovered ciliated cells surface density were increased in aged mouse airways, with a decreased tendency in proximal to the distal direction. Recently, a novel perspective established regarding the characteristics of CS‐induced epithelial reprogramming, including decreased club cells, loss of ciliated cells, and dysfunction in the remaining ciliated cells [8].

The bronchial epithelial cells were abundant with club and ciliated cells. More than 95% of nonciliated cells are club cells that contain club cell secretory proteins (CCSP). Human club cells are virtually absent in the proximal airway epithelium; nevertheless, they increase progressively in distal airways [9, 10, 11]. The proximal‐distal axis of club cells' dynamic process and its function have attracted increasing attention. Club cell biological functions are diverse, including anti‐inflammatory role, anti‐oxidative stress activities, immune modulation, and induce alveolar regeneration. Recently, several studies of aging‐related lung disease have focused on human distal bronchial epithelial cells, providing unique insights into COPD disease treatments [9, 12, 13, 14, 15].

COPD has a complex pathogenesis and is poorly understood. In previous study, we performed single‐cell RNA sequencing (scRNA‐seq) on human COPD lung tissue and found senescent club cells [16]. Additionally, a novel subtype of club cells was recognised in COPD, with functions different from those previously reported [17, 18, 19]. Hence, we focused on club cells in the present study.

COPD is strongly associated with accelerated lung aging. Klotho (KL), a classical anti‐aging protein, is predominantly expressed in the kidney distal tubular epithelial cells and brain choroid plexus. KL‐deficient mice showed premature senile features, and KL was reportedly reduced in the lung tissues, muscles, and peripheral blood in COPD patients. Furthermore, KL was reduced in COPD animal models. Similar results were observed when bronchial epithelial cells were incubated with cigarette smoke extract (CSE) [20, 21]. Club cells are belong to subtype of bronchial epithelial cells; however, KL and club cells have not been noticed before.

Homozygous mutant klotho (KL^**−/−**^) mice had pulmonary emphysema and diverse aging phenotypes at an early stage [22, 23]. Thus, we established whole‐body deletion of KL using CRISPR/cas9 technology. In this study, we aimed to investigate the potential relationship between KL, club cells, and COPD.

## 2 Materials and Methods

### 2.1 Animals

All procedures were conducted following humane animal care standards with approval from the Nanjing Medical University Ethics Committee (2021‐SR‐335). C57BL/6N and KL knock out mice were obtained from Cyagen Biosciences Inc. (Nanjing, China). Lung tissue about KL^−/−^ and wild type mice were collected for scRNA‐seq, aimed to explore potential mechanisms about lung aging. The control groups were subjected to room air (RA), and the experiment groups were placed to CS/Ozone, after which they were sacrificed. Next, BALF and lung tissue were collected, which were used in the follow‐up experiments.

### 2.2 Cell Culture

The human bronchial epithelial cells (BEAS‐2B cell line) and human club cells (H441 cell line) were obtained from the Chinese Academy of Sciences (Shanghai, China). All cell lines were authenticated using short tandem repeat profiling.

### 2.3 CS‐Induced COPD Animal Model

Male C57/B6 mice and heterozygous mutant Klotho (KL^+/−^) mice, with average age of 6–8 weeks, living in a specific pathogen‐free environment. The control groups were exposed to RA (*n* = 8–10 each). The experimental groups were placed in CS environment generated by burning Marlboro cigarettes [containing nicotine (0.8), tar (10 mg), and carbon monoxide (11 mg)] (5 days per week, twice per day, lasted for 8 or 24 weeks). Next, All mice were sacrificed, BALF and lung tissues were collected.

### 2.4 Senescence‐Associated β‐Galactosidase (SA‐β‐Gal) Staining

The SA‐β‐gal staining kit was purchased (Beyotime, C0602) and staining was performed based on the manufacturer's protocol. Briefly, the cell samples or frozen lung tissue sections were fixed with 4% formaldehyde for 10 min. Next, the slides were rinsed with PBS and incubated with freshly prepared SA‐β‐Gal staining solution overnight at 37°C. Afterward, the cells or tissue sections were washed with PBS. Finally, the images were captured using a microscope equipped with a digital camera (Olympus, Japan).

### 2.5 Scanning Electron Microscopy (SEM) and Transmission Electron Microscope (TEM)

The mice airways were washed in PBS, cuted into appropriate shape and subjected to 2.5% glutaraldehyde solution at 4°C. Then, the samples were fixed on aluminium stubs. We observed the results on JEOL JSM‐7900F scanning electron microscope and JEOL JEM‐1400Flash Transmission electron microscope. More methods were performanced as described in the online data supplement.

### 2.6 Statistical Analysis

All assays were performed at least in triplicates. Results are expressed as mean ± standard deviation. Comparisons were performed using the student's *t*‐test between two groups or anova in multiple groups, and *p* < 0.05 was considered statistically significant.

## 3 Results

### 3.1 Lung Tissue Single‐Cell RNA Sequencing Analysis of COPD Patients Revealed Club Cell Heterogeneity

**FIGURE 1:**
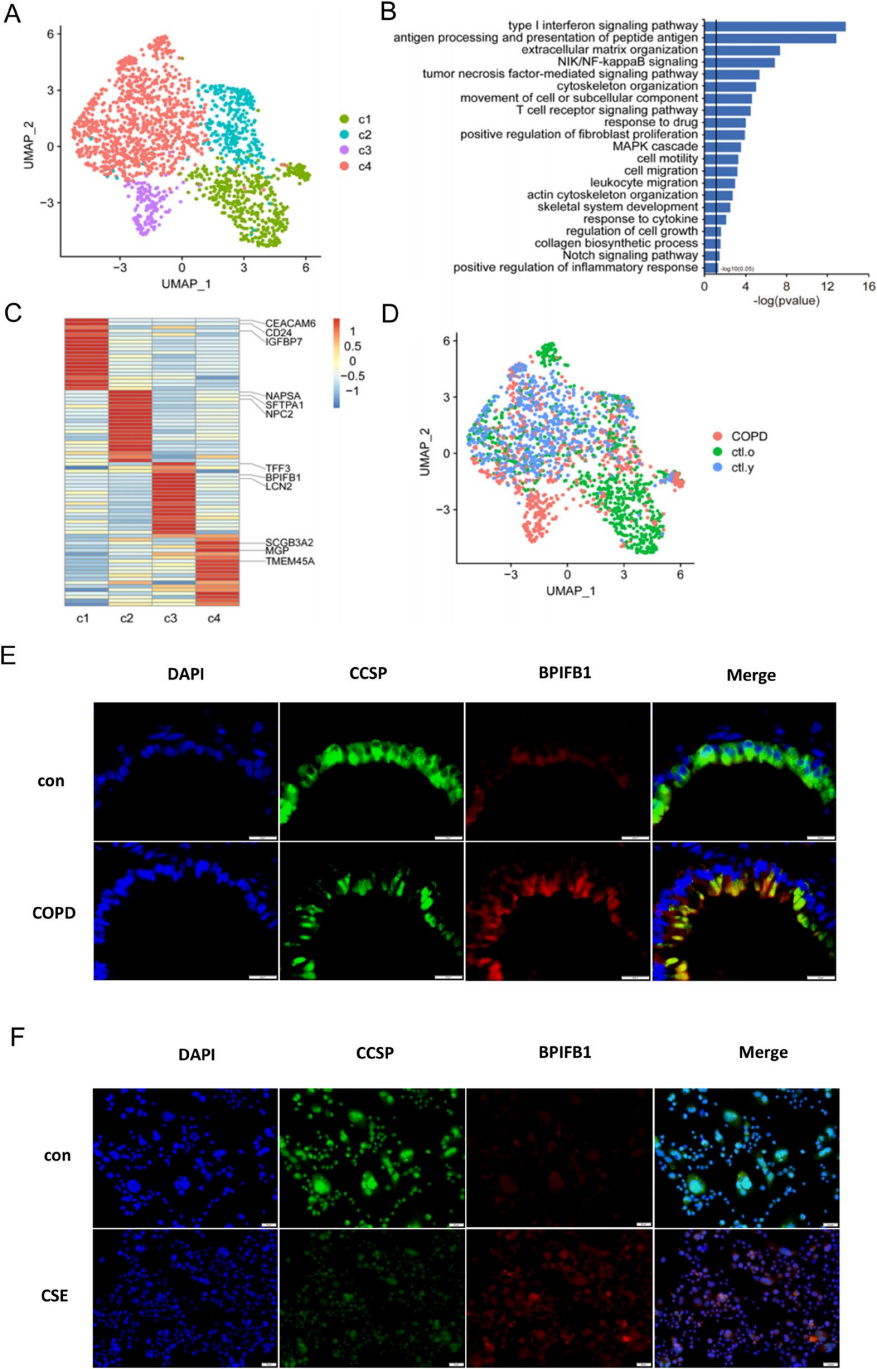
Lung tissue single‐cell RNA sequencing analysis of COPD patients revealed club cell heterogeneity. (A) UMAP plot suggested sub‐clusters of club cells in human COPD lung tissue, including c1, c2, c3 and c4. (B) Bar plots showed the activated pathways involved in the c3 subtype of club cells, mainly including immune and inflammation aspects. (C) Heatmap displaying the functional annotation of the sub‐cluster‐specific expressed genes in different subtypes about club cells. (D) UMAP plot presented that the c3 subtype of club cells mainly originated from COPD lung tissue. (E) Double‐immunofluorescence revealed that BPIFB1 was elevated in club cells, when examined in CS‐induced mice lung tissues. All mice were male, aged 6–8 weeks, *n* = 3–4 per group. DAPI: Blue; green: CCSP; red: BPIFB1. Scale bar = 20 μm. (F) Double‐immunofluorescence revealed that BPIFB1 was elevated in CS‐induced human club cells. Human club cells were cultivated with 5%CSE for 24 h. DAPI: Blue; green: CCSP; red: BPIFB1. Scale bar = 50 μm.

To better understand the major molecular pathological features of various cell types, we collected nine lung tissues (three COPD and six controls) using scRNA‐seq. Notably, club cells were presented with strong relationship with senescence and had four types (c1, c2, c3 and c4) (Figure 1A). The innovative finding was that heterogeneous population of club cells were identified and a population was named as immune‐associated club cells, which abundant with immune‐related changes, such as interferon, antigen processing, and NF‐kB signalling pathways activation (Figure 1B,C). In addition, scRNA‐seq analysis revealed immune‐associated club cells mainly originated from lung tissue (Figure 1D), indicating pulmonary inflammation in COPD were strongly correlate with club cells alteration.

Immunofluorescence staining on CS‐induced COPD animal model and CSE‐incubated with club cells, which revealed enhanced BPIFB1 expression in club cells, consistent with our previous studies (Figure 1E,F). Prior studies have reported that BFIFB1 existed disorders in multiple diseases, including COPD [24, 25]. Air‐liquid interface showed that BPIFB1 could be secreted by human airway epithelial cells [26]. Consequently, we considered that BPIFB1 was a potential marker of immune‐associated club cells.

**FIGURE 2:**
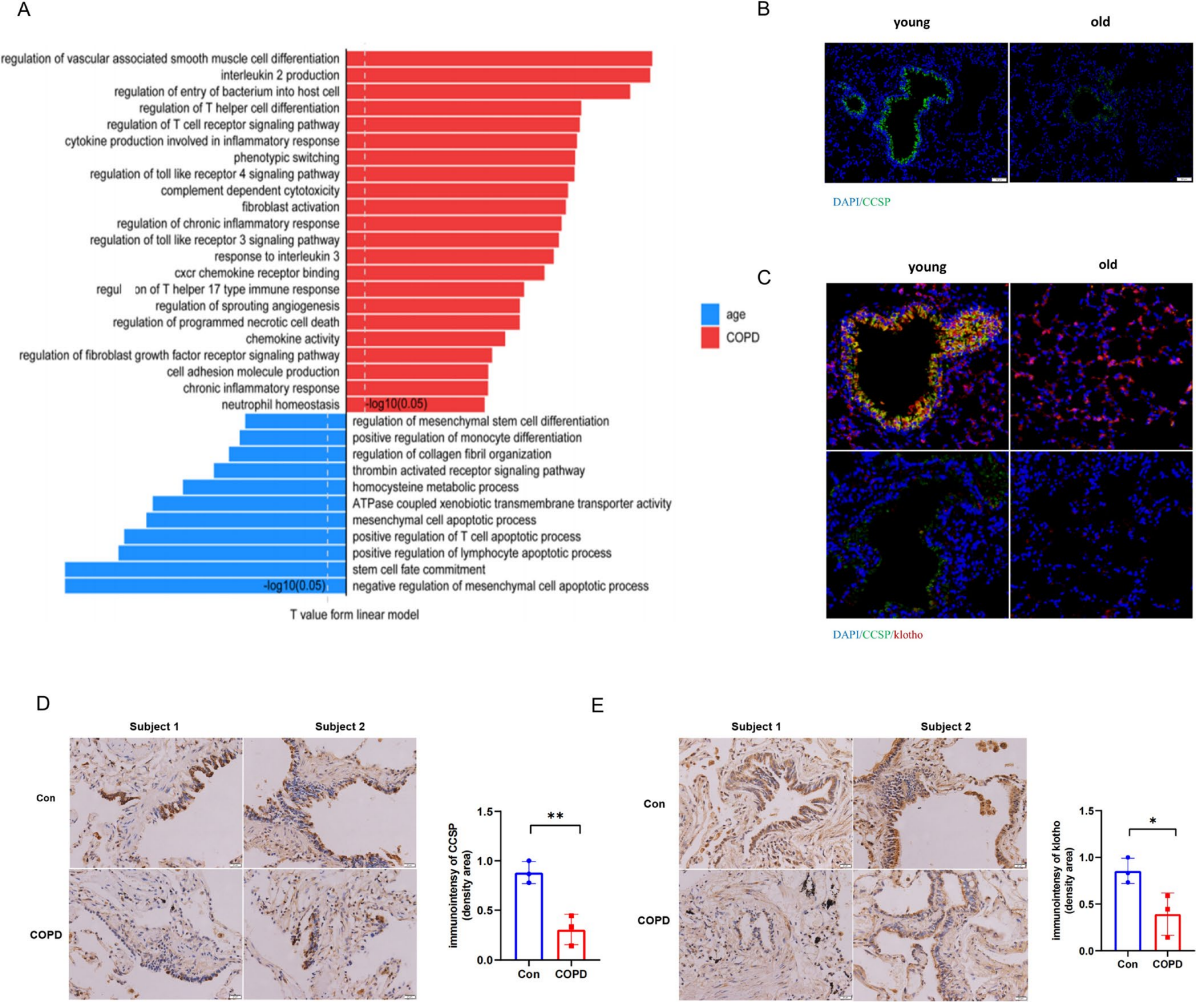
KL and CCSP expressions were downregulated in aged mice lung tissue and COPD patients. (A) Bar plots showed the significant pathways of club cells in aging and COPD conditions. (B) Representative immunofluorescence results revealed CCSP expression was decreased in aged mice. The aged mice were 24 months, and the younger mice were 2 months. Blue: DAPI; green: CCSP. *N* = 3 per group. Scale bar = 50 μm. (C) Representative doubled‐immunofluorescence results indicated KL and CCSP were co‐expressed and reduced in aged mice. The aged mice were 24 months, and the younger mice were 2 months. Blue: DAPI; green: CCSP; red: KL. *N* = 3 per group. Scale bar = 50 μm. (D) Representative immunohistochemistry images and it's quantitative results revealed CCSP was reduced in COPD patients, when compared with non‐COPD patients. *N* = 3–5 per group. Scale bar = 20 μm. (E) Representative immunohistochemistry images and it's quantitative results revealed KL was reduced in COPD patients, when compared with non‐COPD patients. *N* = 3 per group. Scale bar = 20 μm.

Interestingly, we also noticed club cells possessed different changes in different conditions (aging and COPD disease). For instance, club cells mainly focused on inflammation and immune‐related activities in the COPD group. Yet, club cells in the aging condition were abundant with stem cell commitment, homocysteine metabolic process, and mesenchymal cell apoptotic process (Figure 2A). These results proved club cells have participated significant roles in inflammation.

### 3.2 KL and CCSP Were Downregulated in COPD Patients, Aged Mice and Chronic Cigarette Smoke (CS)‐induced COPD Model

We investigated KL and CCSP expressions in aged mice. Immunofluorescence of 24‐months mice exhibited that they were co‐expressed and reduced in the aged mice (Figure 2B,C). Next, we observed target genes distribution in human lung tissue. Moreover, KL and CCSP were both decreased in COPD patients by immunohistochemistry, and the difference were statistically significant (Figure 2D,E).

**FIGURE 3:**
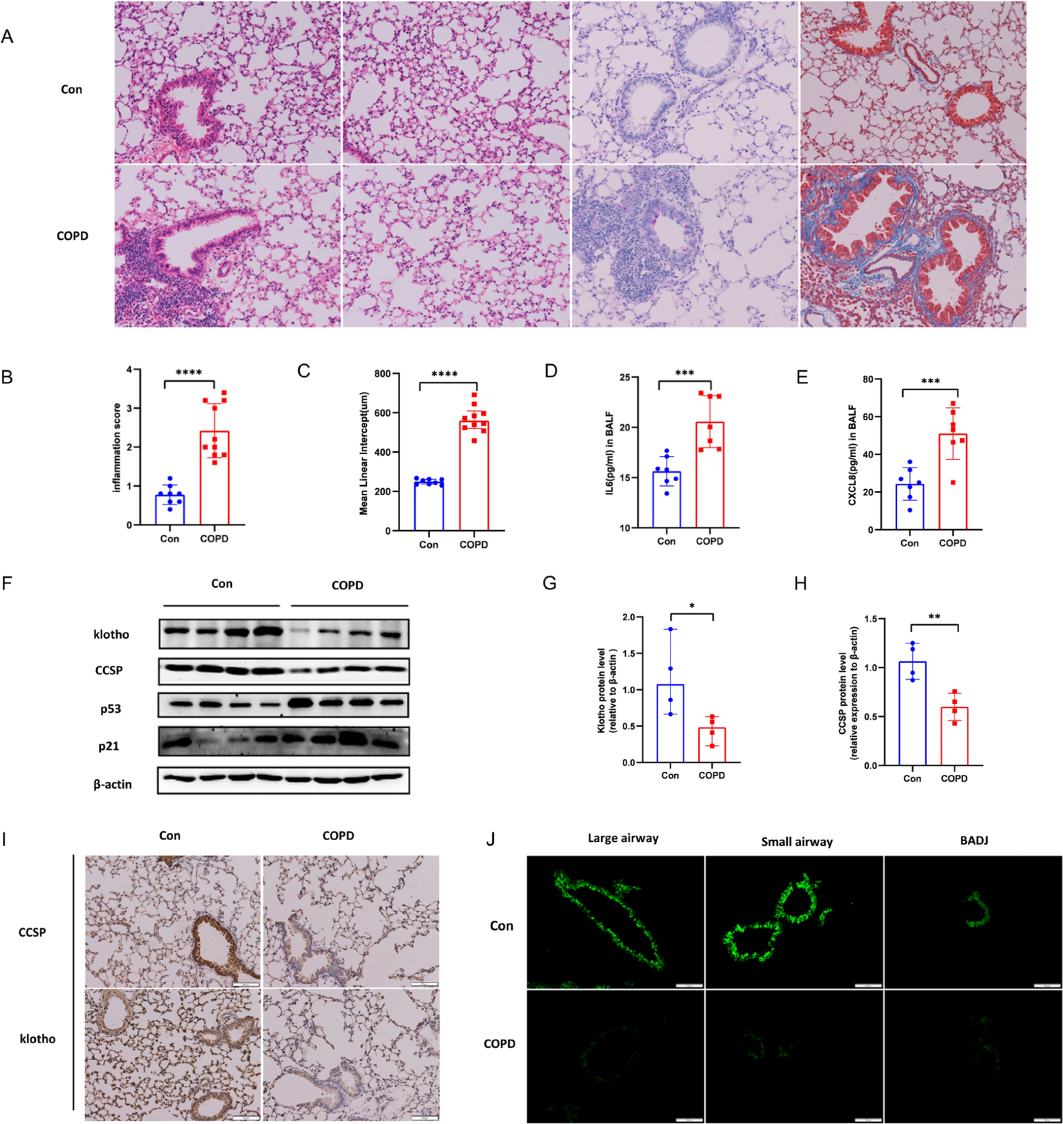
KL and CCSP were decreased in CS‐induced COPD lung tissue in mice. (A) Representative images of HE, Masson trichrome, and PAS staining were showed. Male C57BL/6 mice aged 6–8 weeks were divided into the experimental or control groups, among them, the experimental groups were exposed to CS environment for 24 weeks and the control groups were exposed to RA (*n* = 8–10 per group). Scale bar = 50 μm. (B) Inflammation score was evaluated among COPD animal model and it's control. (C) MLI was calculated among COPD animal model and it's control. (D) IL6 among BALF was assessed among COPD animal model and it's control. (E) IL8 among BALF was assessed among COPD animal model and it's control. (F) KL, CCSP, and senescence‐related markers (p53 and p21) were performed using western blotting. The experimental groups were assessed by CS‐induced COPD animal model, and the control groups were placed to RA (*n* = 4 per group). All data shown represent the mean ± SEM. *p < 0.05, **p < 0.01, ***p < 0.001, and ^****^ *p* < 0.0001. (G) KL relative protein levels were quantified (*n* = 4 per group). (H) CCSP relative protein levels were quantified (*n* = 4 per group). (I) KL and CCSP proteins were analysed using immunohistochemistry. The experimental groups were assessed by CS‐induced COPD animal model, and the control groups were placed to RA (*n* = 3–4 per group). Scale bar = 100 μm. (J) CCSP expression was observed by immunofluorescence in COPD animal model, the locations including large airways, small airways, and bronchiole‐alveolar duct junction (BADJ). The experimental groups were assessed by CS‐induced COPD animal, and the control groups were placed to RA (*n* = 8–10 per group). Blue: DAPI; green: CCSP. Scale bar = 200 μm.

Based on CS‐induced COPD animals, lung inflammation, mean linear intercept (MLI), tissue fibrosis, mucus secretion, and bronchoalveolar lavage fluid (BALF) inflammation levels were assessed. Mice were higher in pulmonary inflammation, fibrosis andand airspace, in response to CS‐exposure environment (Figure 3A–E). The analysis of lung tissue, including western blot, immunohistochemistry and immunofluorescence results, revealed elevated in age‐related markers in lung tissue of COPD mice. Besides, KL and CCSP were presented with a lower level, and the differences between groups were statistically significant (Figure 3F–J).

In vitro experiments, human club cells were cultured with CSE. The results showed that increased senescence of club cells and downregulated CCSP expression. In addition, when human bronchial epithelial cells BEAS‐2B were cultivated with CSE, western blot demonstrated that KL and CCSP expressions were decreased, and KL exhibited a protective role in maintaininll barrier function (Figure S1).

Summarily, KL and CCSP expressions were reduced in aged mouse lung tissue, COPD patients, CS‐induced COPD model, and bronchial epithelial cells incubated with CSE.

### 3.3 KL Deficiency Further Decreased CCSP Expression and Augmented the Senescence of Lung Tissue in Mice

Homozygous mutant klotho (KL^−/−^) mice exhibited multiple phenotypes, which were similar to those observed during human aging, including develop spontaneous emphysema. Consequently, we established KL^−/−^ mice to investigate whether KL will influence CCSP (Figure S2).

**FIGURE 4:**
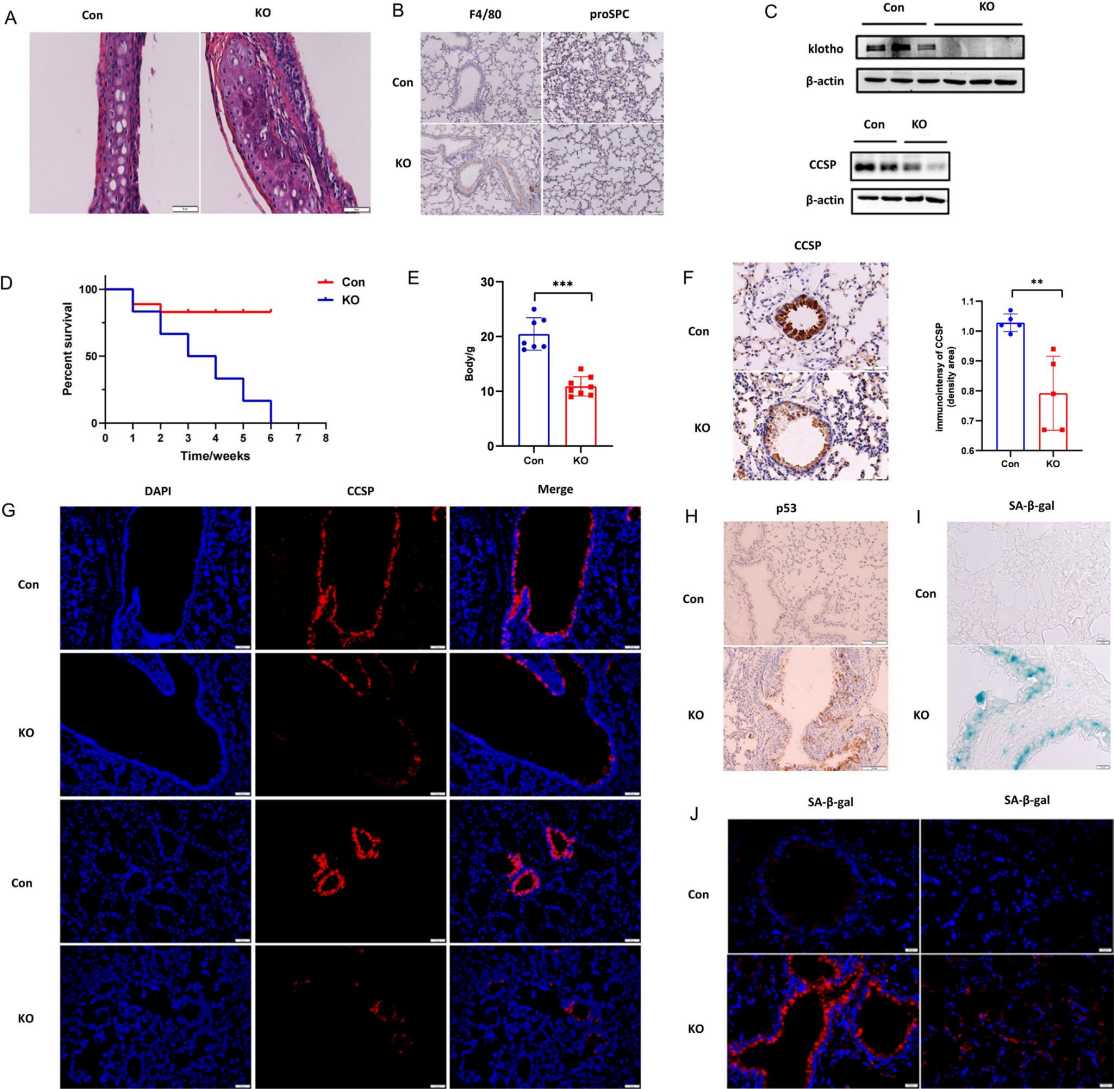
KL deficiency further decreased CCSP expression and augmented the senescence of lung tissue in mice. (A) Representative H&E staining revealed that airway inflammation enhanced in KL^**−/−**^ mice, when compared with Wild‐Type mice. All mice were males, aged 5–8 weeks. Scale bar = 50 μm. (B) Representative immunohistochemistry staining revealed KL^−/−^ lung were strongly associated with monocyte cell infiltration and AT2 reduction. KL^**−/−**^ and Wild‐Type mice were males, aged 5–8 weeks (*n* = 3 per group). Scale bar = 50 μm. (C) Levels of KL and CCSP were analysed using western blot, based on KL^−/−^ and Wild‐Type lung tissue. (D) KL^**−/−**^ and Wild‐Type mice's overall survival were assessed. All mice were males, aged 5–8 weeks (*n* = 7—10 per group). (E) KL^**−/−**^ and Wild‐Type mice's body weight were assessed. All mice were males, aged 5–8 weeks (*n* = 7–10 per group). All data shown represent the mean ± SEM. *p < 0.05, **p < 0.01, ***p < 0.001 and ^****^ *p* < 0.0001. (F) Representative immunohistochemistry staining showed CCSP was reduced after KL knockout, and the difference between groups was statistically significant. All mice were KL^−/−^ or Wild‐Type mice, aged 5–8 weeks (*n* = 5 per group). Scale bar = 50 μm. All data shown represent the mean ± SEM. *p < 0.05, **p < 0.01, ***p < 0.001 and ^****^ *p* < 0.0001. (G) Immunofluorescence revealed that CCSP was reduced after KL knockout. KL^**−/−**^ and Wild‐Type mice were males, aged 5–8 weeks (*n* = 3 per group). DAPI: Blue; red: CCSP. Scale bar = 50 μm. (H) Representative immunohistochemistry stain of p53 increased after KL knockout, KL^**−/−**^ and Wild‐Type mice were males, aged 5–8 weeks (*n* = 3 per group). Scale bar = 100 μm. (I) Representative SA‐β‐gal staining suggested bronchus were increased with senescence after KL knockout, KL^**−/−**^ and Wild‐Type mice were males, aged 5–8 weeks (*n* = 3–5 per group). Scale bar = 10 μm. (J) Immunofluorescence of SA‐β‐gal staining revealed KL^−/−^ lung enhanced with senescence. Blue: DAPI; red: SA‐β‐gal. Scale bar = 50 μm.

As Figure 4A–E described, KL^−/−^ mice presented with higher senescence features, including emphysema, shortened life span, underweight, and aggravated lung inflammation. Therefore, we confirmed KL^−/−^ mice were created successfully. CCSP expression was further examined and decreased when KL was eliminated (Figure 4F,G). Furthermore, we checked KL^−/−^ lung senescence status via aging indicators, such as p53 and SA‐β‐gal staining. These results confirmed that CCSP was reduced and lung aging increased in KL^−/−^ mice (Figure 4H–J). Conclusively, KL can regulate CCSP levels and the senescence of lung tissue in mice.

### 3.4 KL Directly Regulated Club Cells Differentiate Into Ciliated Cells in Mice

**FIGURE 5:**
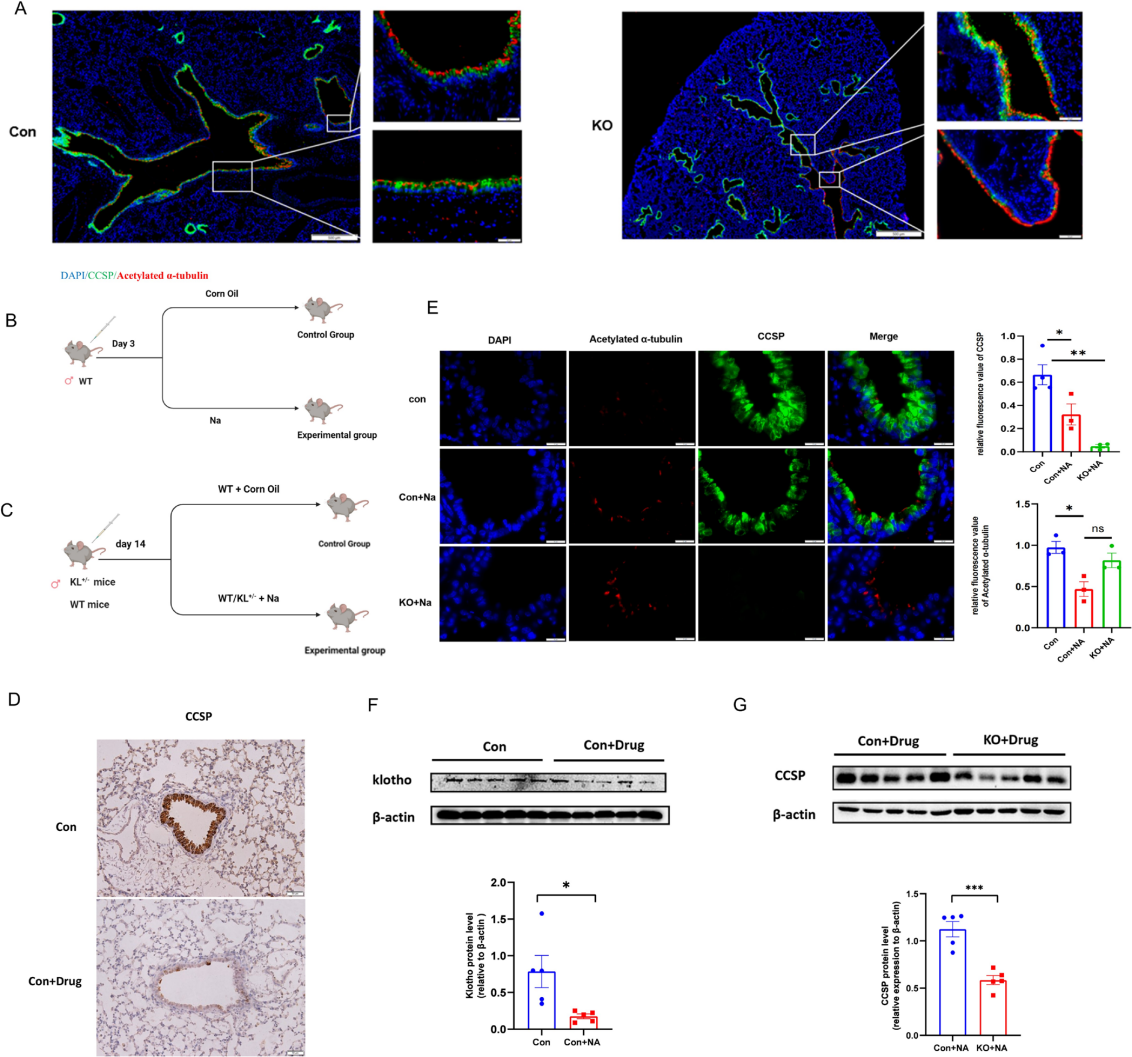
KL directly regulated club cells differentiation into ciliated cells in mice. (A) Representative double‐immunofluorescence showed more ciliated cells appeared where club cells disappeared in KL^−/−^ lung. KL^**−/−**^ and Wild‐Type mice were males, aged 5–8 weeks. Blue: DAPI; Green: CCSP; Red: Acetylated α‐tubulin. Scale bar = 50 μm. (B) The pattern diagram of Na‐induced club cell injury model. All mice were males, aged 6–8 weeks, were injected with Na or corn oil at 200–250 mg/kg at initial dose, lung tissues were collected after 3 days (*n* = 3–5 per group). (C) The pattern diagram of Na‐induced club cell injury models. All mice were males, aged 6–8 weeks, were injected with Na or corn oil at 200–250 mg/kg at initial dose, lung tissues were collected after 14 days (*n* = 7–10 per group). (D) Representative immunohistochemistry of CCSP showed the majority of club cells disappeared in Na‐induced lung tissue after 3 days. Scale bar = 50 μm. (E) Representative double‐immunofluorescence revealed that KL^+/−^ combined with Na‐induced club cell injury possessed higher proportions of ciliated cells. KL^**+/−**^ and Wild‐Type mice were males, aged 5–8 weeks. All mice were injected with Na or corn oil at 200–250 mg/kg at initial dose, lung tissues were collected after 14 days (*n* = 7–10 per group). The quantitative results related to CCSP and acetylated α‐tubulin were analysed. Blue: DAPI; green: CCSP; red: Acetylated α‐tubulin. Scale bar = 20 μm. (F) KL expression was analysed using western blotting, when Na‐induced lung was utilised. All mice were males, aged 6–8 weeks. All mice were injected with Na or corn oil at 250–300 mg/kg at initial dose, lung tissues were collected after 14 days (*n* = 5 per group). *p < 0.05, **p < 0.01, ***p < 0.001 and ^****^ *p* < 0.0001. (G) CCSPexpression was measured using western blot, when Na‐induced lung injury was utilised in KL^+/−^ mice. All mice were males, aged 6–8 weeks. All mice were injected with Na or corn oil at 200–250 mg/kg at initial dose, lung tissues were collected after 14 days (*n* = 5 per group). *p < 0.05, **p < 0.01, ***p < 0.001 and ^****^ *p* < 0.0001.

Pulmonary stem cell studies are debatable and complicated. Generally, progenitor cells would proliferate and differentiate in order to repair the damaged tissue [27]. In our previous study, scRNA‐seq revealed club cells could differentiate into type II alveolar epithelial cells (AT2), type I alveolar epithelial cells (AT1), and ciliated cells [16]. To explore KL's effect on epithelial cells, different cells status were observed by immunofluorescence. More ciliated cells appeared where club cells disappeared in KL^−/−^ lung (Figure 5A, Figure S3). Therefore, we drew preliminary conclusions that KL could regulate club cells differentiation fate.

Subsequently, KL knockout mice combined with club cell injury model were utilised, to further validate the potential relationship between KL and club cells differentiation. In view of KL^**−/−**^ mice life span shortened, and their numbers were limited, KL^+/−^ mice became our choice. KL^+/−^ mice had been reportedly more than 50% reduction in KL [28]. As determined by immunohistochemistry, the majority of club cells in mice were depleted after they were treated with 200–250 mg/kg naphthalene (Na) by intraperitoneal injection (Figure 5B–D).

**FIGURE 6:**
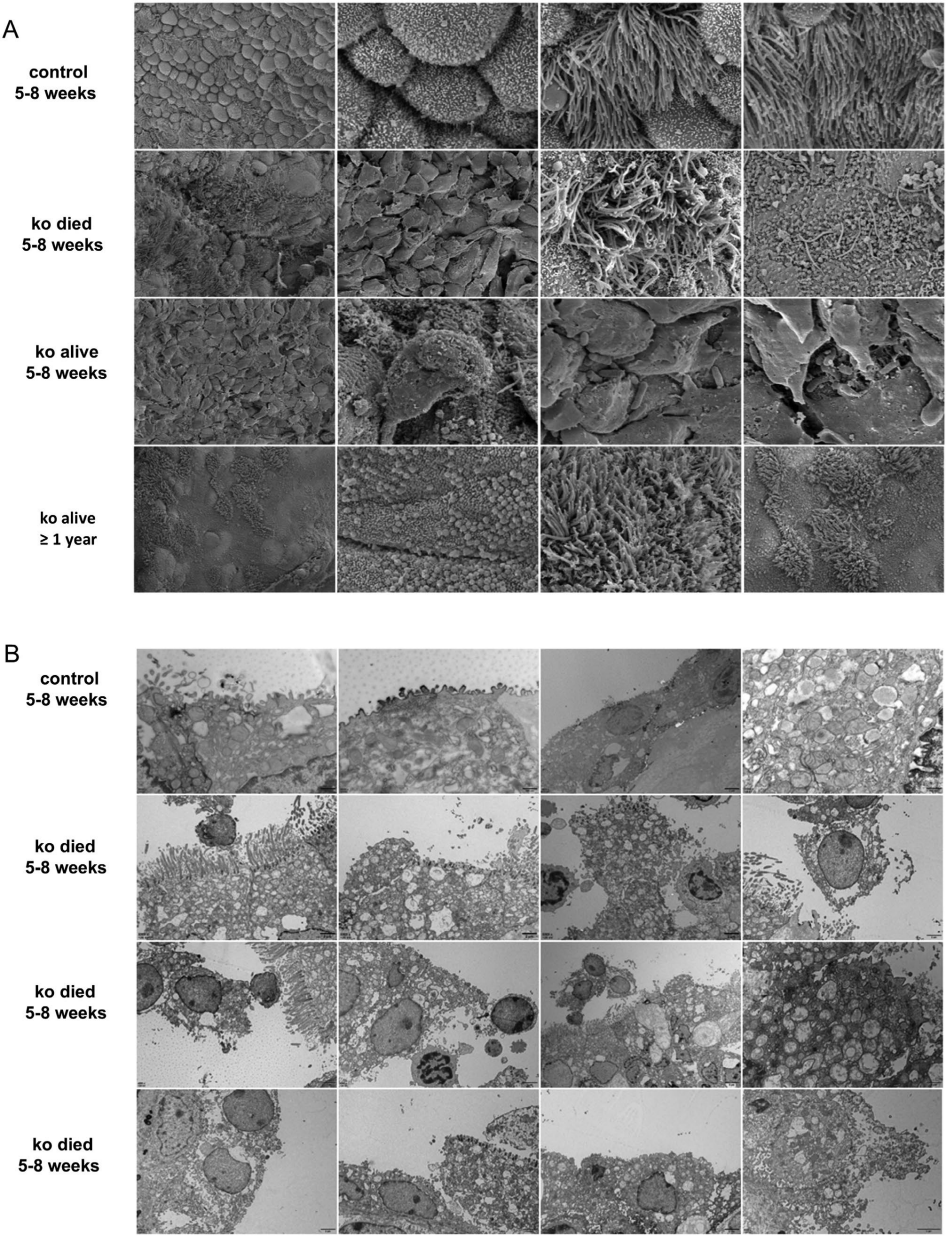
KL deficiency more susceptible to suffer club cell alterations. (A) SEM of KL^−/−^ mice showed club cells microvilli lossed, ciliated cells dysfunction. The mice trachea were collected and divided into four groups, including control group (aged 5–8 weeks), KL died group (aged 5–8 weeks), KL alive group (aged 5–8 weeks), and KL alive (more than 1 year). The scales varied in size from 1 μm to 2 μm, with the majority measuring at 1 μm. (B) TEM assessment of the airway epithelium in mice, including control and KO groups. The higher proportion of apoptosis and mitochondrial damage were observed in KO mice. The scales were range 500 nm from 5 μm, and most of them were 2 μm.

Then, we assessed the proportion of club cells versus ciliated cells. KL^−/−^ mice showed increased ciliated cells where club cells disappeared. Club cells were reduced in Na‐induced lung tissue, and the difference between Wild‐Type mice and KO groups were statistically significant (Figure 5E). Wild‐Type mice with club cell injury presented with lower KL level, and KL^+/−^ with Na‐induced club cell injury model were downregulated in CCSP (Figure 5F,G). Additionally, we observed club cells status by SEM. KL^−/−^ and Wild‐Type mice airways were washed and collected, including ko died mice (5–8 weeks), ko alive mice (5–8 weeks), and ko alive mice (more than 1 years). Interestingly, club cell appearance and numbers were altered, club cells microvilli lossed and ciliated cells dysfunction (Figure 6A). In addition, Transmission electron microscope (TEM) were utilised and club cell apoptosis phenomenon was recognised in ko mice (Figure 6B). All together, we concluded KL could regulate club cells differentiation into ciliated cells in mice.

### 3.5 KL Deficiency Aggravated Club Cell Senescence in CSE‐Induced Pulmonary Inflammation by Neddylation and MLN4924 Treatment Could Reverse Senescence

**FIGURE 7:**
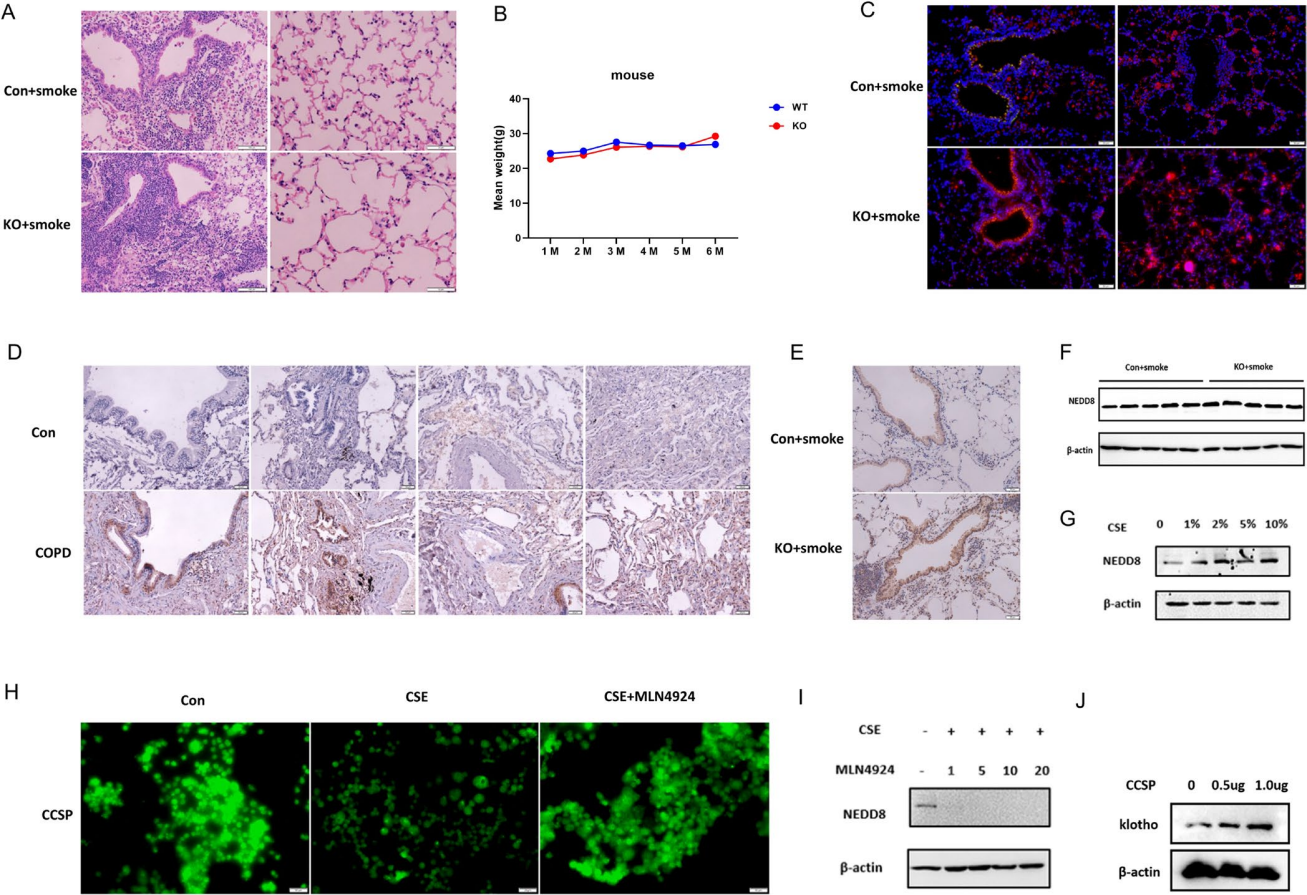
KL deficiency was susceptible to club cell senescence in CSE‐induced pulmonary inflammation by neddylation and MLN4924 treatment could reverse senescence. (A) Representative H&E stain based on CS‐induced lung in mice. KL^+/−^ and Wild‐Type mice were males, aged 6–8 weeks, were exposed to 24‐weeks CS (*n* = 10–12 per group). Scale bar = 50 μm. (B) The weight change of KL^+/−^ and Wild‐Type mice during different times. (C) Representative immunofluorescence stain based on CS‐induced lung in mice. KL^+/−^ and Wild‐Type mice were male, aged 6–8 weeks, were exposed to 24‐weeks CS (*n* = 3–4 per group). Blue: DAPI; green: CCSP; red: NEDD8. Scale bar = 50 μm. (D) Neddylation increased in COPD human lung tissue based on immunohistochemistry. *n* = 3 per group. (E) KL deficiency aggravated neddylation in CS‐induced COPD animal model. (F) Western Blot confirmed aggravated neddylation in CS‐induced COPD animal model. (G)Western Blot proved the expression of NEDD8 was increased with CSE in dose‐dependent manner. (H) Representative immunofluorescence stain showed MLN4924 had a rescue effect in CSE‐induced CCSP reduction. (I) Western Blot confirmed that MLN4924 had a rescue effect in CSE‐induced NEDD8 increased. The concentration of MLN4924 were 0, 1, 5, 10, 20 μM in respectively. (J) Western Blot showed that KL was raised with CCSP expression. CCSP was over‐expressed in human bronchial epithelial BEAS‐2B cells using plasmid.

Neddylation was a focused areas about post‐translational modifications. Dysregulation of neddylation or deneddylation have been regarded as a potential strategy in age‐related diseases. To further determine the relationship between club cell senescence and neddylation, KL^+/−^ and WT mice were exposed to chronic CS environment. As it showed, KL deficiency enhanced lung inflammation, club cells senescence and activated in NEDD8, even though the weight was no significant difference among two groups (*p* < 0.05) (Figure 7A–C, Figure S4). Immunohistochemistry was also suggested COPD patients were higher in neddylation (Figure 7D). In CS‐induced COPD animal model, KL deficiency mice were observed higher NEDD8 level (Figure 7E,F). Besides, Western Blot proved NEDD8 protein increased with CSE concentrations (Figure 7G). Surprisingly, CCSP reduction and NEDD8 upregulation could be inhibited after MLN4924 treatment (Figure 7H,I). In addition, when CCSP was over‐expressed, KL was increased in a dose‐dependent manner (Figure 7J).

Altogether, we confirmed that KL deficiency aggravated club cell senescence in CSE‐induced lung inflammation by neddylation and MLN4924 could be deemed as an effective method to reverse senescence.

### 3.6 Identification of hnRNPA2/B1 Was the Key Molecule in KL‐Regulated Club Cell Senescence

**FIGURE 8:**
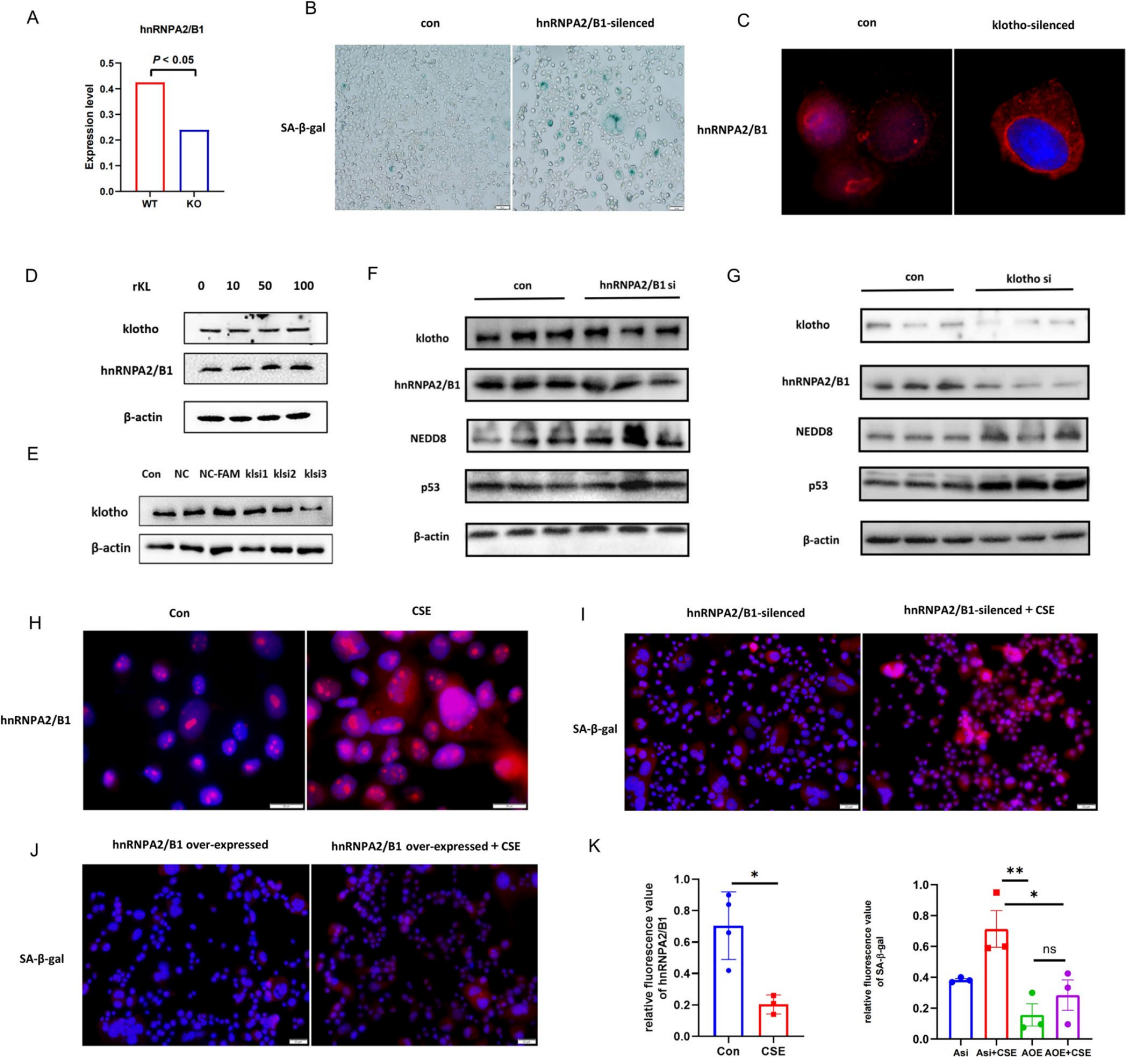
Identification of hnRNPA2/B1 was the key molecule in KL‐regulated bronchial epithelial cell senescence. (A) scRNA‐seq of lung tissue from KL‐/‐ and WT mice revealed that hnRNPA2/B1 was downregulated in club cell when KL deficiency, and the difference was statistically significant. *N*=1 per group. (B) SA‐β‐gal staining results indicated that the hnRNPA2/B1‐silenced group exhibited increased club cell senescence. The intervention time was 72 h. Scale bar = 50 μm. (C) Representative immunofluorescence showed that KL could regulate subcellular localization of hnRNPA2/B1 and its expression. DAPI: Blue; red: hnRNPA2/B1. Scale bar = 20 μm. (D) Western Blot proved that hnRNPA2/B1 expression existed a linear relationship with KL. Human bronchial epithelial cells were exposed to rKL for 24 h. rKL concentration were 0, 10, 50 and 100 ng/mL. (E) Representative western blot results proved that KL was silenced. The intervention time was 72 h. (F, G) Western Blot proved hnRNPA2/B1 was a downstream molecule of KL‐mediated club cell senescence. Club cells were utilised with different conditions, including KL‐silenced and hnRNPA2/B1‐silenced, and the expression of protein (KL, hnRNPA2/B1, NEDD8, p53) were examined. The intervention time was 72 h. (H) Representative immunofluorescence showed that hnRNPA2/B1 nucleocytoplasmic transport was observed in CSE‐exposed club cells. Club cells were cultivated with 5%CSE for 24 h. DAPI: Blue; red: hnRNPA2/B1. Scale bar = 50 μm. (I) Representative immunofluorescence showed hnRNPA2/B1‐silenced in club cells would aggravate cellular senescence. Club cells were cultivated with 5% CSE and siRNA for 24 h. DAPI: Blue; red: SA‐β‐gal. Scale bar = 10 μm. (J) hnRNPA2/B1—overexpressed in club cells would ameliorate cellular senescence. Club cells were cultivated with 5% CSE and plasmids for 24 h. DAPI: Blue; red: SA‐β‐gal. Scale bar = 50 μm. (K) The quantitative results for total hnRNPA2/B1 and SA‐β‐gal were analysed.

To explore the underlying mechanism about club cell senescence, we collected KL knockout mice lung tissues and using scRNA‐seq. It was noteworthy that heterogeneous ribonucleoprotein A2/B1 (hnRNPA2/B1) was the significant differential gene in club cell when KL deficiency (Figure 8A). Emerging evidence reported that hnRNPA2/B1 was increased in patients with lung cancer, and regarded it as a prognostic marker. However, the role of hnRNPA2/B1 in COPD is still unclear. We speculated hnRNPA2/B1 played a critical role in KL‐regulated club cell senescence. Firstly, the downregulated of hnRNPA2/B1 was confirmed in vitro and SA‐β‐gal staining results proved higher senescent cells in hnRNPA2/B1‐silenced group (Figure 8B). HnRNPA2/B1 distribution did not change in normal status, interestingly, hnRNPA2/B1 was mainly distributed in the cytoplasm when KL was silenced (Figure 8C). Besides, the expression of hnRNPA2/B1 enhanced with increasing recombinant KL (rKL) concentrations (Figure 8D). Moreover, hnRNPA2/B1 expression was reduced in KL^−/−^ mice and chronic CS‐exposed KL^+/−^ mice. Ozone‐induced lung tissue injury is an another classical COPD animal model, yet western blot confirmed that there was no significant change about hnRNPA2/B1 (Figure S5). To sum up, we concluded KL could regulate hnRNPA2/B1 sublocation and expression, moreover, cigarette smoke was vital factor to influence hnRNPA2/B1 expression.

Then, klotho‐silenced and hnRNPA2/B1‐silenced were established, aimed to observe the relationship between KL and hnRNPA2/B1 in further. Western Blot showed hnRNPA2/B1 was reduced in KL‐silenced, moreover, p53 and NEDD8 were increased (Figure 8E–G, Figures S6 and S7). Thus, KL was deemed to upstream of hnRNPA2/B1. Next, we checked hnRNPA2/B1's subcellular localization and cellular senescence based on immunofluorescence staining. hnRNPA2/B1 nucleocytoplasmic transport was observed in CSE‐exposed club cells. hnRNPA2/B1 total expression was downregulated, and the difference was statistically significant. In addition, hnRNPA2/B1‐silenced group in club cells proved enhanced in senescence and hnRNPA2/B1 over‐expressed had a reverse effect, and the difference were statistically significant (Figure 8H–K, Figure S6). In summary, we concluded hnRNPA2/B1 was the key molecule in KL‐regulated club cell senescence.

## 4 Discussion

We elucidated the relationship between KL and the small airway epithelial cells. Briefly, our data analysed club cell heterogeneity/senescence in COPD patients using scRNA‐seq, and we proved KL could regulate club cell senescence and differentiation.

Senescence and CS are predominant risk factors for COPD development. Our data observed senescent phenotype of club cells in different animal models, including KL^**−/−**^ mice, aged mice, and COPD mice. Furthermore, club cells possessed aging features when KL deficiency, and it suggested that KL could regulate club cells senescence in further. We noticed the phenomenon that club cell senescence could cause chronic pulmonary inflammation, the viewpoint was consistent with Sagiv [29].

Except for club cell senescence, its specific traits, such as heterogeneity, were investigated. In this study, immune‐associated club cells were identified and BPIPB1 was regarded as the potential marker. In addition, we discovered KL could regulate club cell differentiate into ciliated cells. However, the activation or inactivation pathways during the turnover period remain unclear. Kopan [30] detected that canonical notch signalling was required in club cells differentiation process. Additionally, lineage tracing of scgb1a1^+^ club cells revealed that the majority of club cells in the bronchioles could self‐renew and generate ciliated cells. Similarly, people found that club cells located in the trachea could give rise to ciliated cells and contribute to tracheal repair; however, club cells located in the trachea could not self‐renew extensively. Researchers believed different epithelial progenitor cells maintained trachea, bronchioles, and alveoli cells [31]. In addition, Purushothama [32] detected club cells could dedifferentiate into basal cells. Despite these discoveries on club cells, many areas requiring exploration exist.

Understanding terminal bronchioles and their function is vital to exploring COPD mechanisms. A study discovered that human terminal bronchioles existed a special cell style, that can regenerate AT2 [33, 34]. Recently, Samir et al. [9] detected that human distal airway cell disorder exists and recognised region‐specific cellular heterogeneity along the bronchoalveolar axis.

CCSP was the most widely expressed protein in club cells. Several studies showed that lack of CCSP would increase macrophage and neutrophil levels, when CCSP knockout mice were exposed to CS environment. Undoubtedly, club cells exert a crucial protective role in COPD; however, little is known regarding the exact mechanism of club cells reduction. In our study, we found CCSP decreased after mice were exposed to chronic CS, and the result was consistent with prior studies. Additionally, CS‐induced KL^**+/−**^ mice demonstrated that KL could enhance lung inflammation and the senescence of club cells. At present, views about club cells reduction mechanisms have been inconsistent. Some scientists considered that club cell and CCSP decrease were related to changes in small airway epithelial cell epigenetics [35]. People have identified that glucocorticoids and retinoic acid could stimulate CCSP expression. Moreover, club cells are vulnerable to injury from CYP450 enzyme metabolism, resulting the phenomenon about CCSP decrease. CS and toxic metabolites have similar damage effects on club cells; however, in other lung diseases, such as asthma and interstitial lung disease, CCSP reduction still exists. Altogether, it indicated that CCSP reduction may be involved in various regulatory processes [36, 37, 38, 39].

Besides club cells, variant club cells (vCCs) were also recognised in previous studies. vCCs can resist Na‐induced cell damage because it does not contain cytochrome P450. vCCs could express CCSP protein and were primarily located at the distal bronchus, near the neuroepithelial bodies, and BADJ. vCCs can reportedly self‐renew and produce normal club and ciliated cells. In our experiment, we did not distinguish club cells or vCCs.

scRNA‐seq about KL knockout mice revealed that hnRNPA2/B1 was downregulated in club cell. To explore whether KL regulates CCSP through hnRNPA2/B1, we assessed hnRNPA2/B1 expression in different COPD animal models. Western blotting indicated that hnRNPA2/B1 was downregulated in the CS group and remained stable in the ozone group. Moreover, human bronchial epithelial BEAS‐2B cells and club cells were examined hnRNPA2/B1 expression via CSE, incubated with rKL. Then, hnRNPA2/B1, KL, NEDD8 and p53 were assessed based on western blot. To sum up, we supported that hnRNPA2/B1 was the key molecule in KL‐regulated club cell senescence, and neddylation of club cell was a crucial factor contributing to hnRNPA2/B1 reduction. hnRNPA2/B1 expression was distributed broadly. hnRNPA2/B1 had pro‐proliferative and anti‐apoptotic roles in pulmonary arterial smooth muscle cells. Ruffenach [40] demonstrated that inhibite hnRNPA2/B1 was a potential approach to control pulmonary arterial hypertension. In our research, we confirmed target hnRNPA2/B1 is a good therapeutic strategy for COPD disease.

Conclusively, we described the COPD chronic inflammation mechanism through scRNA‐seq, COPD‐related animal models and KL knockout mice. Certainly, our article has some shortcomings. One limitation of this study is that we were unable to observe club cells's differentiation fate by primary human small airway epithelial cells and the tracer mouse model. Additionally, we didnt't clearly explain the regulatory relationship between KL, hnRNPA2/B1 and neddylation. In our subsequent research, we will focus on addressing these issues.

## 5 Conclusions

The senescence and regeneration process about pulmonary bronchial epithelial stem cells were complicated. Our data supported KL could regulate club cell senescence and differentiation, which contribute to CCSP reduction. When CS stimulates small airway epithelium cell, KL deficiency aggravates lung inflammation, club cell senescence and dysfunctional of ciliated cell. Targeting Neddylation might be a promising strategy to reverse lung aging and club cell senescence. These results provide a mechanism about COPD‐linked lung inflammation.

## Author Contributions

**Min Li:** data curation, formal analysis, investigation, writing – original draft. **Bo Chen:** conceptualization, funding acquisition, writing – review and editing. **Sibo Sun, Kai Wang, Yu Wang:** data curation, investigation, writing – review and editing. **Jianqing Wu:** conceptualization, funding acquisition, project administration, writing – review and editing. All authors approved the final manuscript.

## Ethics Statement

For human and mice studies, written informed consent was received from participants.

Prior to inclusion in the study and the study was approved by Nanjing Medical University.

## Conflicts of Interest

The authors declare no conflicts of interest.

## Supporting information


Figure S1.

Figure S2.

Figure S3.

Figure S4.

Figure S5.

Figure S6.

Figure S7.

**Data S1.** Supporting Information.

## Data Availability

All original data can be obtained from the authors upon reasonable request.
