# Supplementary figures and images for: Klotho Regulates Club Cell Senescence and Differentiation in Chronic Obstructive Pulmonary Disease
Source: Cell Prolif. 2025 Feb 10;58(7):e70000. doi: 10.1111/cpr.70000 (PMC12240633; doi:10.1111/cpr.70000)

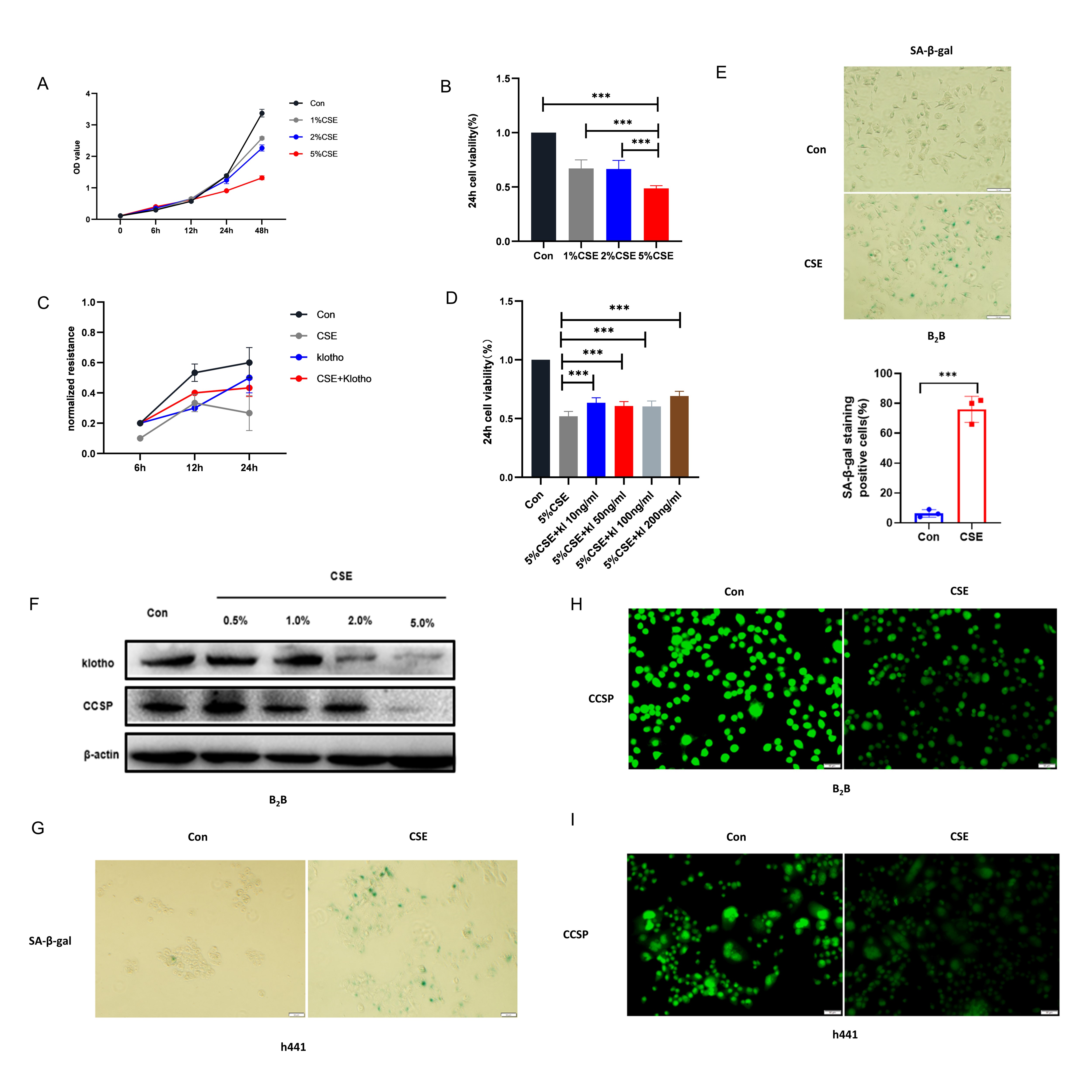

Supplement: Supplementary file 1 — Figure S1. [file CPR-58-e70000-s002.jpg]

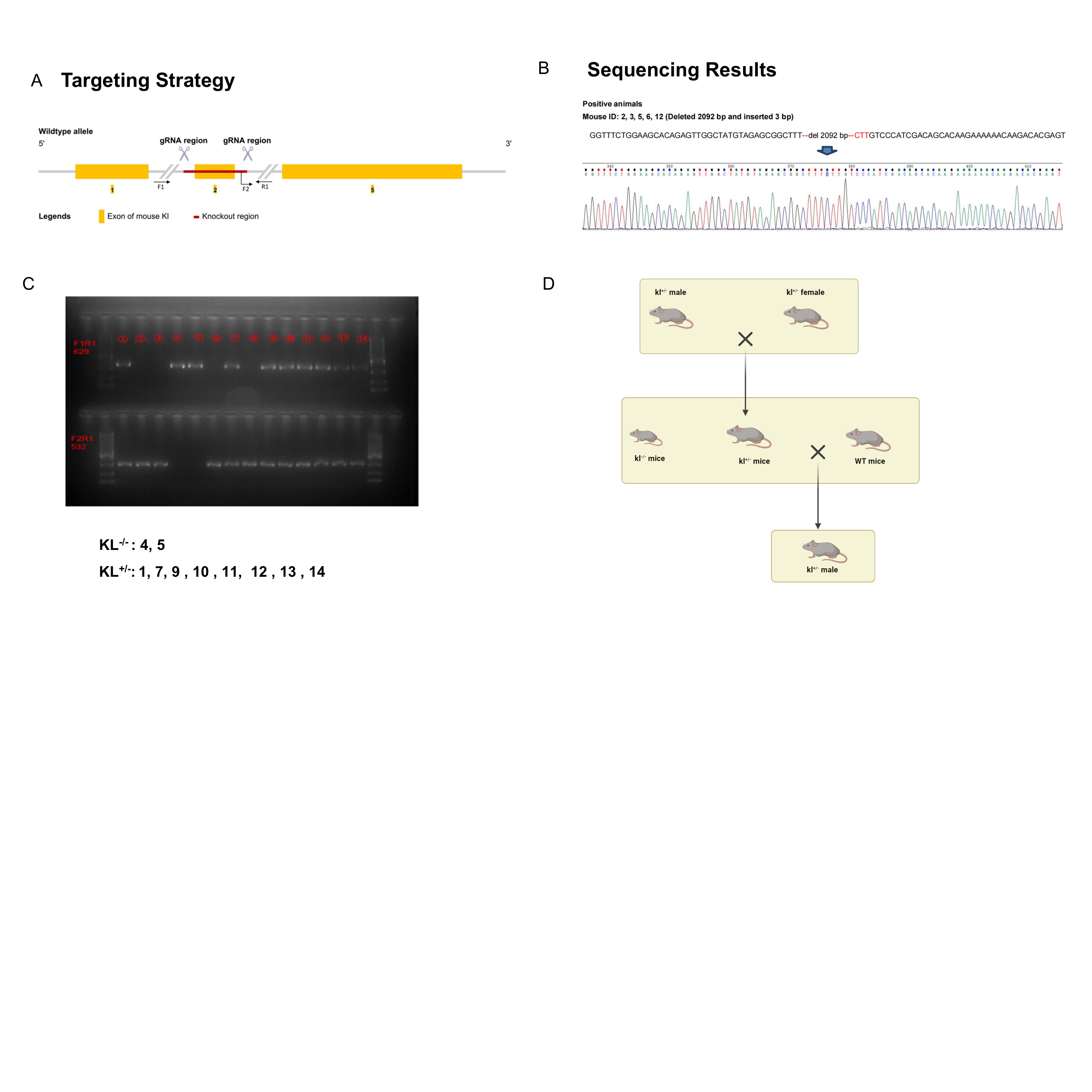

Supplement: Supplementary file 2 — Figure S2. [file CPR-58-e70000-s001.jpg]

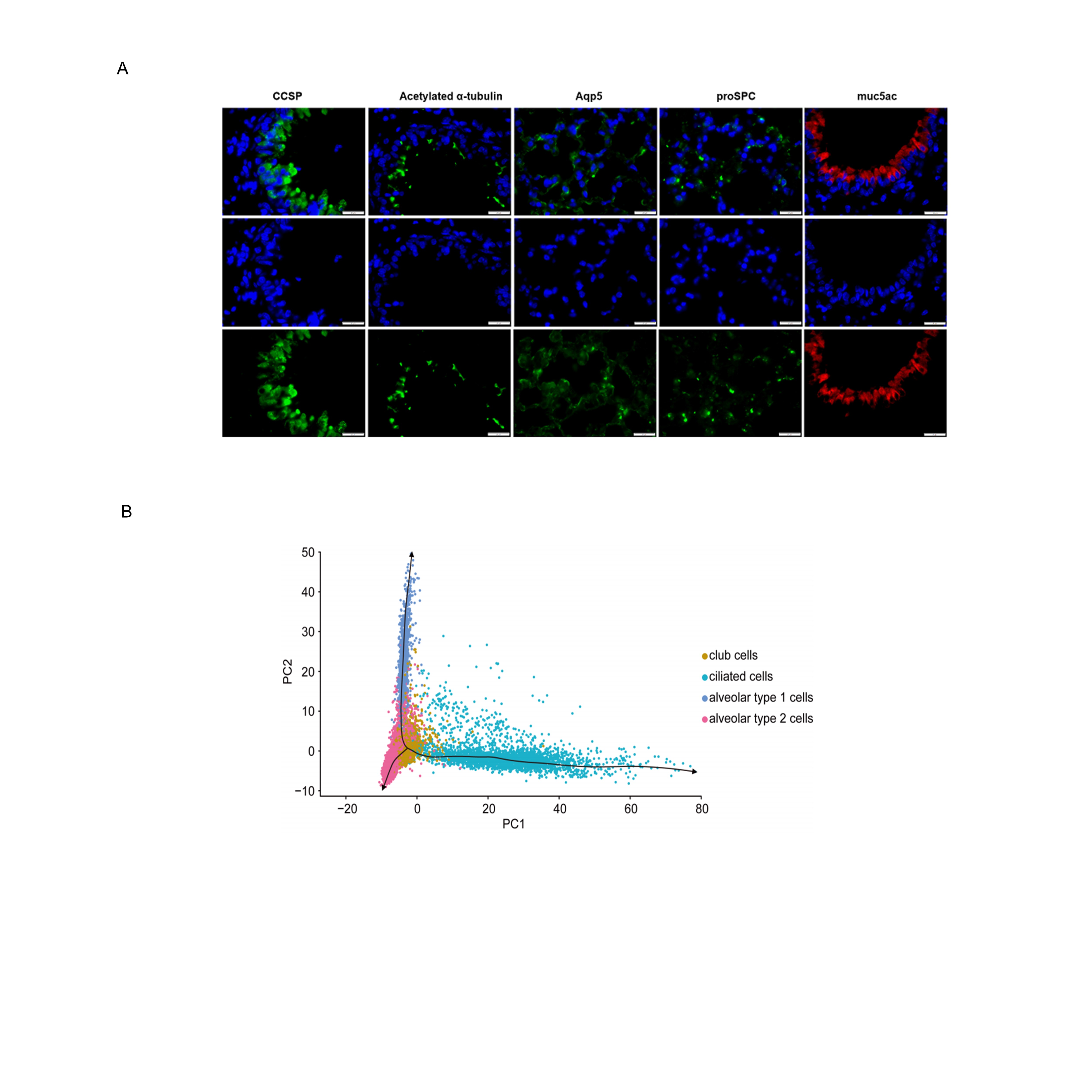

Supplement: Supplementary file 3 — Figure S3. [file CPR-58-e70000-s004.jpg]

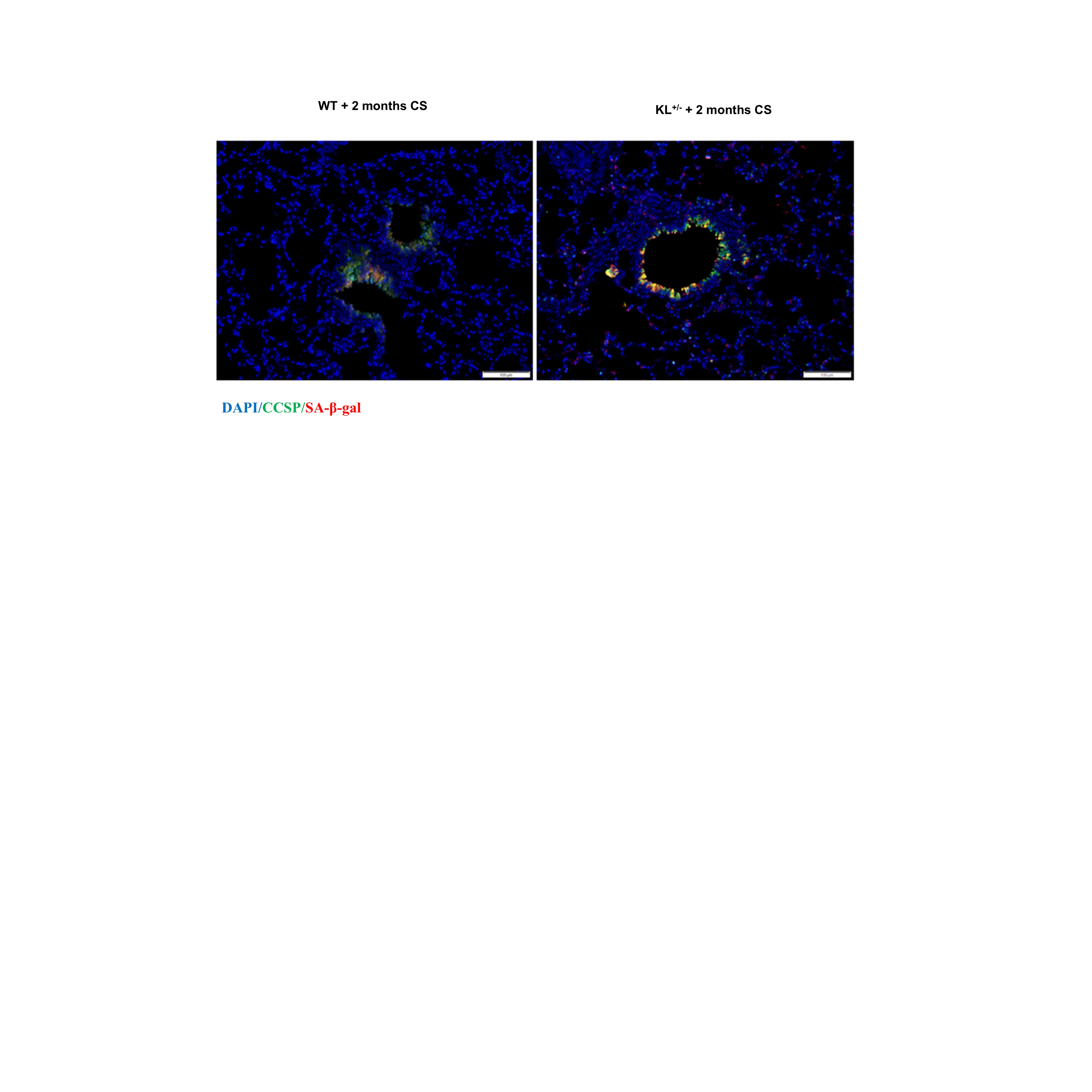

Supplement: Supplementary file 4 — Figure S4. [file CPR-58-e70000-s006.jpg]

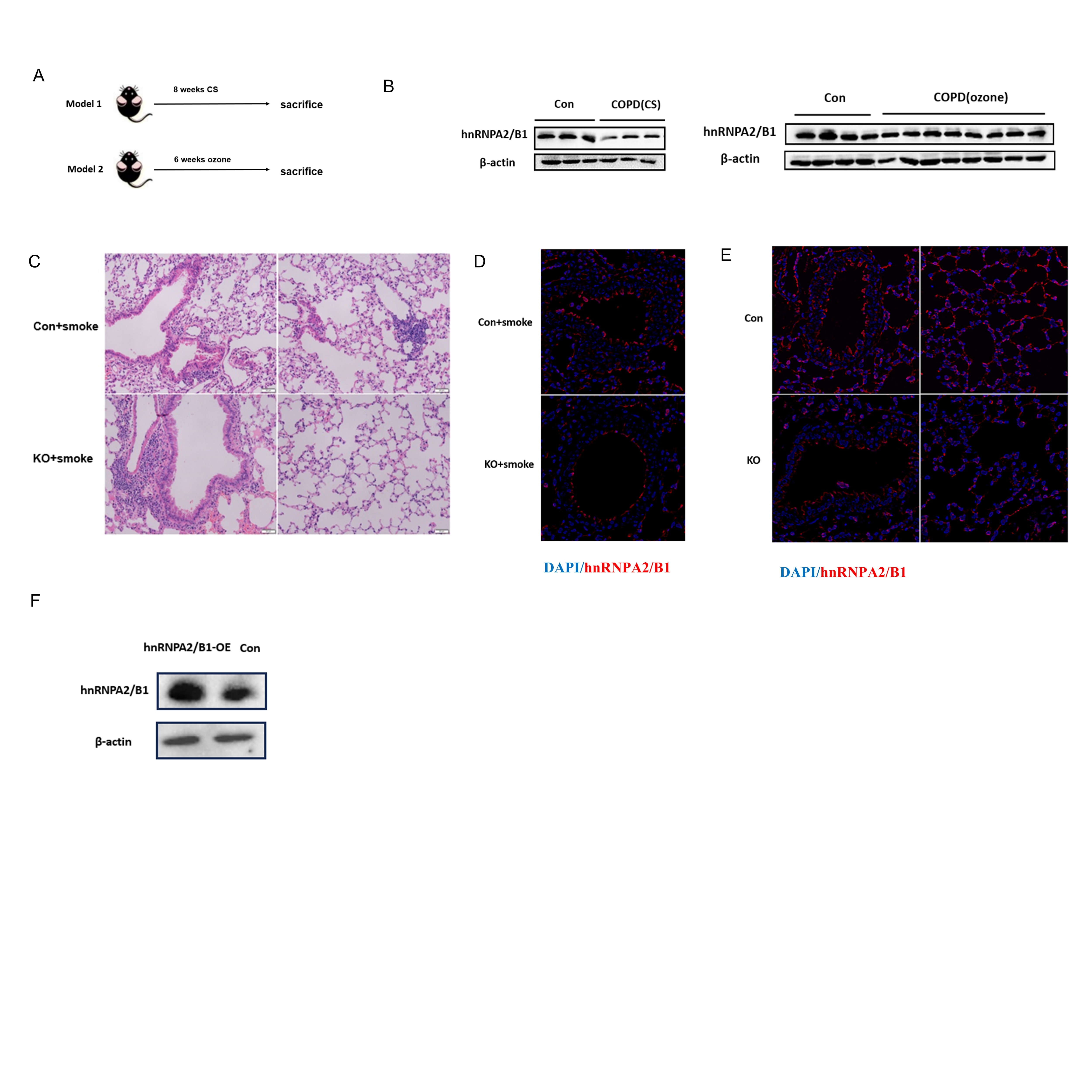

Supplement: Supplementary file 5 — Figure S5. [file CPR-58-e70000-s008.jpg]

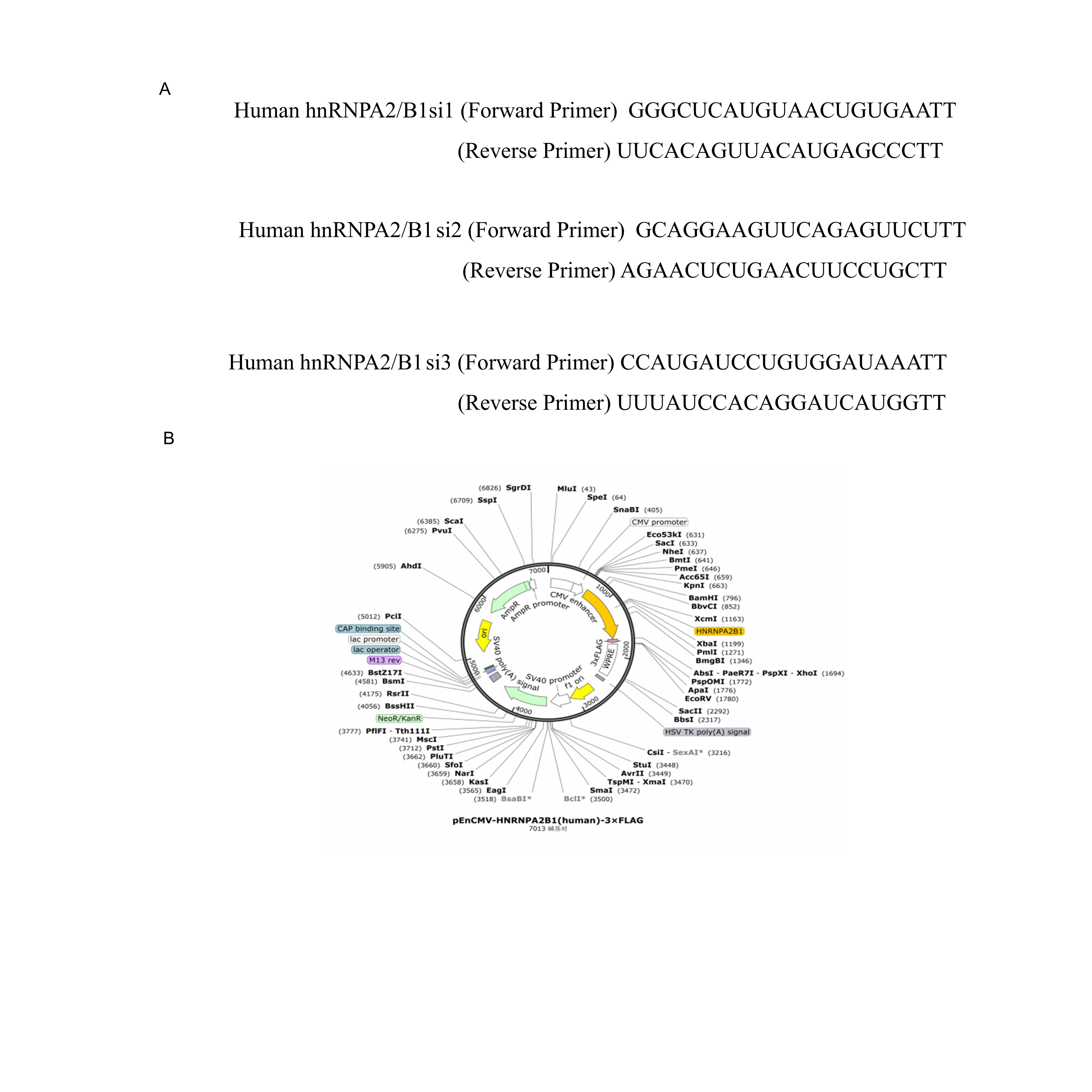

Supplement: Supplementary file 6 — Figure S6. [file CPR-58-e70000-s003.jpg]

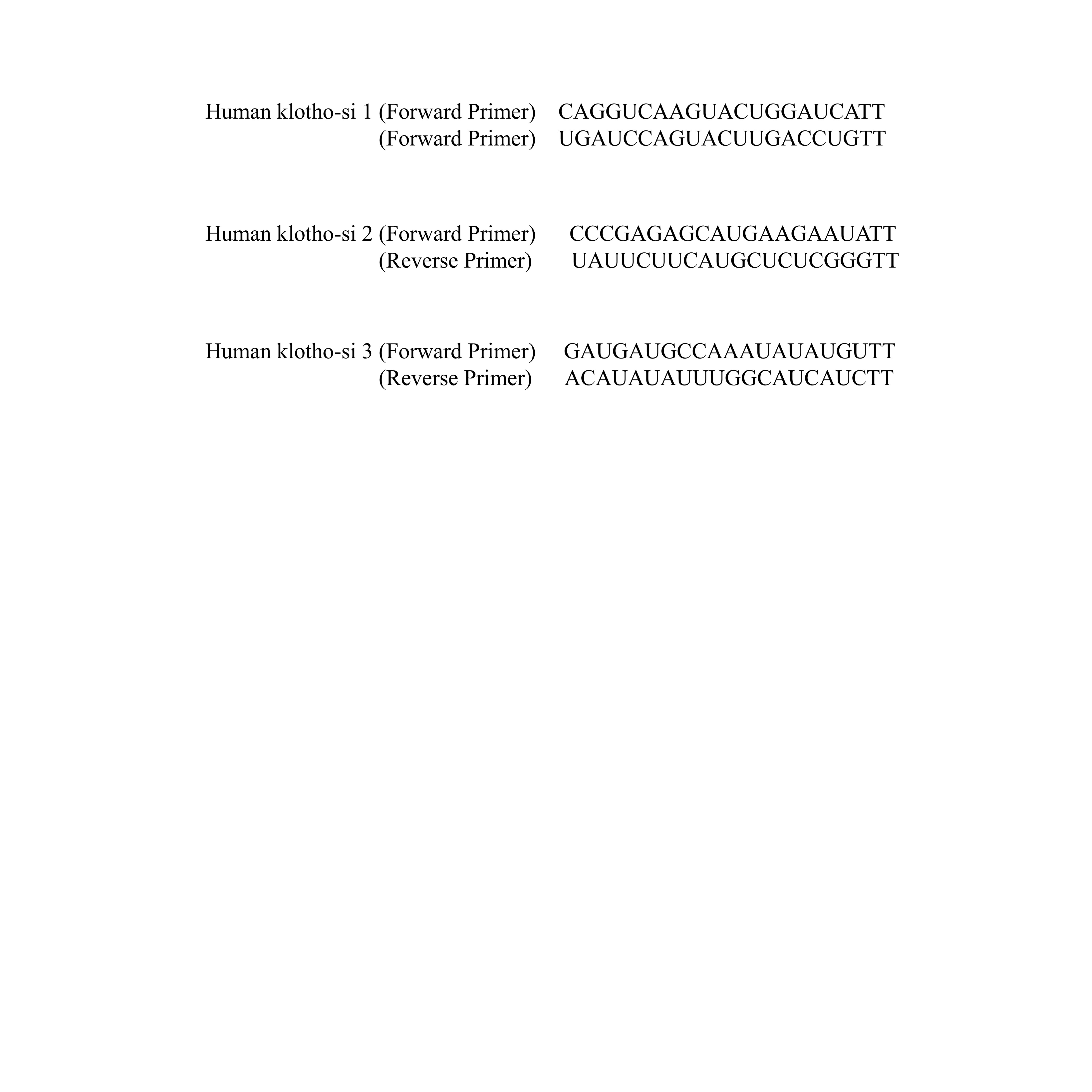

Supplement: Supplementary file 7 — Figure S7. [file CPR-58-e70000-s007.jpg]
