# Supplementary material for: Klotho Regulates Club Cell Senescence and Differentiation in Chronic Obstructive Pulmonary Disease
Source: Cell Prolif. 2025 Feb 10;58(7):e70000. doi: 10.1111/cpr.70000 (PMC12240633; doi:10.1111/cpr.70000)
Supplement: Supplementary file 8 — Data S1. Supporting Information. [file CPR-58-e70000-s005.docx]

**Supplementary**

## Klotho regulates club cell senescence and differentiation ****in chronic obstructive pulmonary disease****

**1.1 Supplementary Methods.........................................................................................................2**

**1.2 Supplementary Figures.....................................................................................................5**

**1.1 Supplementary Methods**

**Immunofluorescence**

Lung tissues and **cells were fixed with 4% paraformaldehyde, permeabilized with 0.3% Triton for 20 minutes, and blocked with 5% normal goat serum in PBS for 1h at room temperature. Next, they were incubated with CCSP antibody (Santa,** sc-365992, **1:50), CCSP antibody (Abcam, Ab213203, 1:100), KL antibody (Abclonal,** A12028, **1:200), BPIFB1 antibody (Invitrogen, MA5-17124, 1:100),** SA-β-Gal **antibody (Invitrogen, A-11132, 1:25),** Acetylated α-tubulin **antibody (Sigma, T7451, 1:1000), hnRNPA2/B1 antibody (Abclonal, A1162, 1:100), muc5ac antibody (Invitrogen, MA5-12178, 1:500), AQP5 antibody (Abcam, Ab78486, 1:500), Prospc antibody (Abcam, Ab3786, 1:500), BPIFB1 antibody (Invitrogen, MA5-17124, 1:100), and** NEDD8 (Abcam, Ab81264, 1:500) **overnight at 4°C. After washing with PBS, the cells were incubated with secondary antibodies, at room temperature for 2 h and washed thrice with PBS. Finally, the nuclei were stained with 4',6-diamidino-2-phenylindole (DAPI) (Beyotime, P0131) for 10 minutes, and the fluorescence images were captured using a confocal microscope (**Olympus**, Japan).**

**Immunohistochemistry (IHC)**

Lung tissues were fixed with 4% paraformaldehyde and embedded with paraffin.

Primary antibodies performed in IHC were KL **antibody** (Abclonal, A12028, 1:200), CCSP **antibody** (Santa, sc-365992, 1:1000), **Prospc antibody (Abcam, Ab3786, 1:500), F4/80 (**Santa, **sc-377009, 1:200)** and NEDD8 (Cell Signaling Technology, 2745S, 1:200).

**Western blotting**

Western blots were performed to verify gene expression in control and COPD groups. Total protein extracts (20 μg) were separated on the appropriate concentration of SDS-PAGE. Finally, the blots were probed with KL antibody (Abcam, Ab214666, 1:1000), CCSP antibody (Santa, sc-365992, 1:1000), p53 antibody (Proteintech, 60283-2-Ig, 1:1000), p21 antibody (Proteintech, 10355-1-AP, 1:1000), hnRNPA2/B1 antibody (Abcam, Ab259894, 1:1000), NEDD8 (Abcam, Ab81264, 1:5000) and β-actin antibody (Proteintech, 66009-1-Ig, 1:10000).

**Plasmids and siRNAs**

Flag-hnRNPA2/B1 were commercially obtained from MiaoLing (Wuhan, China),

and small interfering RNAs (siRNAs) were designed and synthesized by GenePharma (Shanghai, China). All cells were treated according to the manufacturer's instructions.

**Lentivirus construction and infection**

KL silenced BEAS-2B cells were established by lentivirus construction. To construct KL silenced plasmids, human KL cDNA was synthesized and cloned into

pSLenti-U6-shRNA(KL)-CMV-mCherryF2A-Puro-WPRE vector with OBiO (Shanghai, China), and cells were infected using lentivirus vectors carrying specific target genes in the presence of polybrene (10 µg/mL) and screened with 2.5 µg/mL of puromycin (Sigma, USA) for 2 weeks to obtain stable cell lines.

**Elisa assay**

The mouse interleukin (IL)-6 and IL-8 levels in BALF were measured using ELISA kits (RD, USA).

**Ozone-induced COPD animal models**

Mice were divided into two groups: control and ozone. Male C57/B6 mice, with an average age of 6–8 weeks, were chosen. The control groups were exposed to RA, and the experimental groups were placed in ozone (n=8–10 each), which described as the previous protocol [21].

**1.2 Supplementary Figures**

**Supplementary Figure 1.** KL and CCSP expressions were downregulated in CSE-induced human bronchial epithelial cells and was associated with increased senescence.

(A) OD values at 450nm were assessed, when human bronchial epithelial BEAS-2B cells were incubated with different CSE concentrations and times;

(B) Different CSE concentrations for 24 h to observe cell proliferation rates;

(C) Transepithelial resistance ( TEER ) assessment was used to observe cell barrier function, the groups were divided into control, 5%CSE, rKL ( 100 ng/mL ), and CSE combined with rKL;

(D) Different conditions for 24 h to observe cell proliferation rates, the groups were divided into control, 5%CSE, and 5%CSE combined with rKL ( 10 ng/mL, 50 ng/mL,100 ng/mL, and 200 ng/mL ).

(E) SA-β-gal staining was used to assess cell senescence degree, when human bronchial epithelial BEAS-2B cells cultivated with 5% CSE for 72 h. Scale bar = 100 μm.

(F) KL and CCSP expressions were analyzed using western blot, when human bronchial epithelial BEAS-2B cells were cultivated with 0.5% CSE, 1.0% CSE, 2.0% CSE, and 5% CSE for 24 h.

(G) CCSP levels were examined using immunofluorescence, when human bronchial epithelial BEAS-2B cells cultivated with 5% CSE for 24 h. DAPI: blue; Green: CCSP. Scale bar = 20 μm.

(H) SA-β-gal staining was used to assess cell senescence degree, when human club cells H441 cultivated with 10% CSE for 72 h. Scale bar = 100 μm.

(I) CCSP levels were examined using immunofluorescence, when human club cells H441 cultivated with 10% CSE for 72 h. DAPI: blue; Green: CCSP. Scale bar = 20 μm.

**Supplementary Figure 2.** The strategy about KL knock out mice.

(A) Scheme for constructing KL knock out mice.

(B) Sequencing results about KL knock out mice.

(C) Genotyping of offspring from cross breeding.

(D) Scheme for breeding KL knock out mice.

**Supplementary Figure 3.** Different pulmonary cells distribution and differentiation in lung tissues.

(A) Immunofluorescence was applied to observe airway epithelial cells, including club cell marker CCSP and other common cell markers ( Muc5ac, Prospc, Aquaporin 5, Acetylated α-tubulin ).

(B) The trajectory of airway epithelial cells differentiation including club cells, ciliated cells, AT1s, and AT2s.

**Supplementary Figure 4.** After 8 weeks CS, KL^+/-^ mice enhanced club cell senescence, when compared to Wild-Type mice. KL**^+/-^** and Wild-Type mice were males, aged 6–8 weeks ( n=3 per group ). DAPI: blue; Red: SA-β-gal; Green: CCSP. Scale bar = 100 μm.

**Supplementary Figure 5.** hnRNPA2/B1 was the differential genes in KL deficiency mice and downregulated in CS-induced COPD animal model.

(A) Pattern diagram of COPD animal models.

(B) hnRNPA2/B1 protein was examined in CS- and ozone-induced lung in mice, which analyzed by western blotting. C57BL/6 mice aged 6–8 weeks were chosen and divided into CS- and ozone-induced COPD animal models ( n=10—12 and n=8, respectively ).

(C) Representative H&E stain based on CS-induced lung in mice. KL^+/-^ and Wild-Type mice were male, aged 6–8 weeks, were exposed to 8 weeks CS ( n=10—12 per group ). Scale bar = 50 μm.

(D) Representative immunofluorescence showed that hnRNPA2/B1 was decreased in **KL^+/-^** mice, including bronchia and alveolus area. KL**^+/-^** and Wild-Type mice were male, aged 6–8 weeks. All mice were exposed to 8 weeks CS ( n=10—12 per group ). DAPI: blue; Red: hnRNPA2/B1. Scale bar = 100 μm.

(E) Representative immunofluorescence showed that hnRNPA2/B1 was downregulated in **KL^-/-^** mice, when compared to control group. KL**^+/-^** and Wild-Type mice were male, aged 6–8 weeks ( n=3 per group ). DAPI: blue; Red: hnRNPA2/B1. Scale bar = 100 μm.

(F) Representative western blot result confirmed that hnRNPA2/B1 was overexpressed in club cell. Club cells were cultivated with 5%CSE for 48h.

**Supplementary Figure 6.** The strategy of hnRNPA2/B1 silenced and over-expressed.

(A) The primer about hnRNPA2/B1;

(B) The structure of hnRNPA2/B1 plasmid.

**Supplementary Figure 7.** The primer about KL.
